# Safety and economic considerations of argatroban use in critically ill patients: a retrospective analysis

**DOI:** 10.1186/s13019-015-0214-0

**Published:** 2015-02-07

**Authors:** Se-Chan Kim, Nicole Tran, Jens-Christian Schewe, Olaf Boehm, Maria Wittmann, Ingo Graeff, Andreas Hoeft, Georg Baumgarten

**Affiliations:** Department of Anesthesiology and Intensive Care Medicine, University Hospital Bonn, Sigmund-Freud-Str. 25, 53127 Bonn, Germany

**Keywords:** Argatroban, Heparin induced thrombocytopenia, Critical care

## Abstract

**Background:**

Heparin-induced thrombocytopenia (HIT) causes thromboembolic complications which threaten life and limb. Heparin is administered to virtually every critically ill patient as a protective measure against thromboembolism. Argatroban is a promising alternative anticoagulant agent. However, a safe dose which still provides effective thromboembolic prophylaxis without major bleeding still needs to be identified.

**Methods:**

Critically ill patients (*n* = 42) diagnosed with HIT at a tertiary medical center intensive care unit from 2005 to 2010 were included in this retrospective analysis. Patient records were perused for preexisting history of HIT, heparin dosage before HIT, argatroban dosage, number of transfusions required, thromboembolic complications and length of ICU stay (ICU LOS). Patients were allocated to Simplified Acute Physiology Scores above and below 30 (SAPS >30, SAPS <30), respectively. For calculations, patients (n = 19) without previous history of HIT were compared to patients (n = 23) with a history of HIT before initiation of argatroban.

**Results:**

The mean initial argatroban dosage was below 0.4 mcg/kg/min regardless of SAPS score. Maintenance dosage had to be increased in patients with SAPS <30 to 0.54 ± 0.248 mcg/kg/min (*p* >0.05) to achieve effective anticoagulation. No thromboembolic complications were encountered. Argatroban had to be discontinued temporarily in 16 patients for a total of 57 times due to diagnostic or surgical procedures, supratherapeutic aPTT and bleeding without increasing the number of transfusions. A history of HIT was associated with a shorter ICU LOS and significantly reduced transfusion need when compared to patients with no history of HIT. Cost calculation favour argatroban due to increased transfusion needs during heparin administration and increase ICU LOS.

**Conclusion:**

Argatroban can be used at doses < 0.4 mcg/kg/min without an increase in transfusion requirements and at a reduced overall treatment cost compared to heparin.

## Background

In heparin-induced thrombocytopenia (HIT) immunoglobulin G is formed against multimolecular complexes of platelet factor 4 (PF4) and polyanion heparin, which potentially leads to thromboembolic life and limb threatening complications [1,2]. Clinical diagnosis is based on the 4 T score (Thrombocytopenia, Timing, Thrombosis, absence of other explanations) [3,4]. Typically, clinical suspicion of HIT is made by a platelet count drop in the 5–10 days following heparin administration. However, it should be kept in mind that in almost 60% of patients diagnosed with HIT, thrombosis occurs prior to or on the day of significant platelet decrease [5]. Differential diagnoses include EDTA-induced pseudothrombocytopenia, non-immune heparin-associated thrombocytopenia, thrombotic-thrombocytopenic purpura, other drug-induced thrombocytopenias, disseminated intravascular coagulation, acute thrombosis-associated thrombocytopenia and sepsis [6]. Guideline recommendations consist of discontinuation of all heparin, initiation of a non-heparin anticoagulant due to increased risk of thromboembolic events and establishing diagnosis with a serologic assay [7]. PF4 enzyme linked immunosorbent assay (ELISA) detects immunoglobulin G antibodies and is a widely used initial assay. However, this test has poor specificity, which might lead to overdiagnosis and overtreatment of HIT, including increased risk of bleeding [8,9]. Furthermore, costs of alternative anticoagulants imply an additional economic burden [10]. Only a subset of anti-PF4/heparin antibodies activate platelets, which makes certain platelet activation assays, such as the heparin-induced platelet activation assay (HIPAA) and the serotonin release assay (SRA), more specific. However, HIPAA and SRA are restricted to specialized laboratories. Diagnosing HIT in critically ill patients is difficult due to the high incidence of thrombocytopenia in this patient population. The incidence varies and seems to be higher among surgical patients compared to non-surgical patients [11,12].

Argatroban is a direct thrombin inhibitor and is used as an alternative anticoagulation agent in HIT. An initial dosage of 2 mcg/kg/min without a bolus is recommended except for patients with heart failure, liver dysfunction, multiorgan dysfunction syndrome, severe anasarca and early post-cardiac surgery phase. A dose reduction to 0.5 - 1.2 mcg/kg/min is recommended in this patient population, according to the manufacturer. Argatroban follows hepatobiliary elimination. Hence it is the preferred agent in patient in renal failure. But it needs to be closely monitored and dosage must be adjusted in case of hepatic insufficiency [13]. However, the optimal dosage for critically ill patients is still under investigation [14-17]. Even recommended dosages for critically ill patients could lead to blood loss [18]. The aim of this retrospective study was to evaluate the safety of argatroban dosage in critically ill patients, operationalized as number of thromboembolic events (i.e., myocardial infarction, stroke, pulmonary embolism and peripheral arterial occlusion), increased bleeding and need for blood transfusions, as well as mortality and length of ICU stay (ICU LOS). In addition, we compared the overall costs of therapy using argatroban versus heparin.

## Methods

### Ethics statement

Ethical approval for this study (Ethical Committee N° 061/14) was provided by the Ethical Committee Bonn University Hospital, Bonn, Germany (Chairperson Prof. K. Racké) on 26th February 2014. Informed consent was waived and data were analyzed anonymously.

### Patients

This retrospective study included 42 patients (18 female/24 male) in a six year period (2005–2010) who were diagnosed with HIT before or during admission to the ICU and received argatroban during their stay at the surgical ICU at the University Hospital of Bonn, Germany, a tertiary care academic medical center. Initial and maintenance dosage of argatroban and transfusion requirements in these patients were evaluated to assess safe dose ranges for argatroban. Critically ill patients were allocated to Simplified Acute Physiology Score (SAPS) of above 30 (>30) and below 30 (<30) at the time of ICU admission. Changes in SAPS score were documented daily and correlated with argatroban dose. Clinical suspicion of HIT was made if patients had a new onset of thrombocytopenia, recent heparin exposure and/or thromboembolic complications followed by PF4 ELISA. Patients with PF4 ≥ 0.4 OD were defined as HIT positive. Confirmatory HIPAA was additionally performed. Platelet recovery following initiation of argatroban therapy served as a clinical parameter to confirm diagnosis of HIT. Patients´ data and laboratory values were extracted from hospital records and the clinical information system. According to the department´s policy, heparin exposure (e.g. intravenous administration) was discontinued and catheters filled with heparinized solutions were exchanged as soon as clinical suspicion of HIT was raised, while awaiting a confirmatory diagnostic testing. Initial dosage was at the discretion of the responsible physician. One patient received lepirudin before argatroban and one patient danaparoid after argatroban. A third patient received a single oral medication of 100 mg aspirin. The argatroban dosage was adjusted for a target activated partial thromboplastin time (aPTT) 1.5 to 3 times that of the baseline aPTT. The aPTT was routinely monitored twice daily and 4 hrs after dosing changes per ICU policy. Bleeding was assessed by hemoglobin fall and transfusion monitoring of packed red blood cells (PRBC), fresh frozen plasma (FFP) and platelets (i.e., ≧ 2 PRBC in 24 hours and/or a fall in hemoglobin ≧ 2 g/dL). Recognition and diagnosis of thromboembolic complications (e.g., myocardial infarction, stroke, pulmonary embolism and peripheral arterial occlusion) were included in the daily intensive care routine. Renal function was evaluated with serum creatinine, blood urea nitrogen (BUN) and creatinine clearance. Acute kidney failure was treated with continuous renal replacement therapy (CRRT). Liver function tests included bilirubin, liver enzymes (alanine/aspartate aminotransferase (ALT/AST), gamma-glutamyl transferase (GT)) and albumin. Moderate elevation of serum aminotransferase was defined by more than 3 times the upper limit of normal [19]. Respiratory failure was defined as PaO2/FiO2 < 200 mmHg [20]. Furthermore we compared ICU LOS and transfusion requirements for patients with a history of HIT with those patients who received heparin shortly before HIT was suspected (no history of HIT) and argatroban was initiated. The results were used for cost calculations of argatroban versus heparin administration. Calculations were based on current wholesale price of 2.17 EUR per 25.000 IE heparin and 188.12 EUR for 250 mg argatroban. The investigated parameters comprised total daily anticoagulant administration, duration of heparin administration before initiation of argatroban, blood transfusion and ICU LOS were incorporated in the total cost calculation.

### Statistical analysis

All data are reported in mean ± standard error of the mean (SEM). A Student t- test or a Mann–Whitney *U* test was performed where appropriate. Significant differences were considered to exist at *p* < 0.05.

## Results

Patients´ demographics are summarized in Table 1.

**Table 1:** **Demographic data (mean ± SEM,*n*** **= 42, except when marked otherwise)**

Age (years)	61 ± 2.4
Body weight (kg)	84.4 ± 3.2
Body Mass Index kg/m^2^	29.2 ± 1.2
Length of ICU-Stay (days)	15 ± 2.9
history of HIT	6.0 ± 1.2, *n* = 23
no history of HIT	25.8 ± 5.0, *n* = 19
SAPS <30	11 ± 3.0, *n* = 16
SAPS >30	18 ± 4.2, *n* = 26
SAPS score on ICU admission	34 ± 2.2
Sepsis	14 (33%)
Renal replacement therapy	17 (40.5%)
Respiratory failure	31 (73.8%)
Veno-arterial ECMO	1 (2.4%)
Death*	6 (14.2%)

*Death was unrelated to bleeding, thromboembolic complication or argatroban.

### Diagnosis of HIT

Argatroban was initiated in all patients with a history of HIT and suspected HIT which was clinically diagnosed by a history of heparin exposure and thrombocytopenia based on the 4 T score [3,4]. Anticoagulation was initiated when there was no clinical sign of perioperative bleeding and aPTT was in the lower reference range. The average time elapsed from time of ICU admission to starting anticoagulation was 13.22 ± 4.24 hours for patients with a history of HIT and 8.25 ± 3.28 hours in patients without a history of HIT (*p* > 0.05). While 23 (54.8%) patients had a history of HIT, 19 patients (45.2%) with no history of HIT had received heparin for 7.5 ± 1.3 days before HIT was suspected. Exposure to heparin was found in 17 (85%) patients (906.4 ± 186.5 IE/h) up to the day before initiation of argatroban. In the remaining 2 patients, heparin infusion was interrupted more than 24 hours before initiation of argatroban. Based on the 4 T score, there was a high probability (4 T = 6-8) of HIT in 4 (21.1%) patients with no history of HIT, intermediate (4 T = 4-5) in 10 (52.6%) and low probability (4 T≦3) in 5 patients (26.3%). In patients with no history of HIT, PF4 ELISA was positive in 17 cases (94.4%) out of 18 tested patients. In all 5 patients with low HIT probability score, PF4 ELISA and HIPAA were positive, respectively. Mean platelet count was 148.6 ± 19.58 G/L at time of ICU admission.

### Bleeding and thromboembolic complications

**Figure 1:**
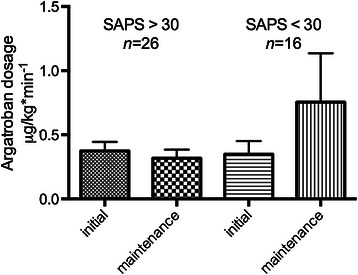
**Initial - and maintenance dosage (mean ± SEM) of argatroban (mcg/kg/min) in critically ill patients depending on SAPS-score.***n* = 42, *p* < 0.05.

**Figure 2:**
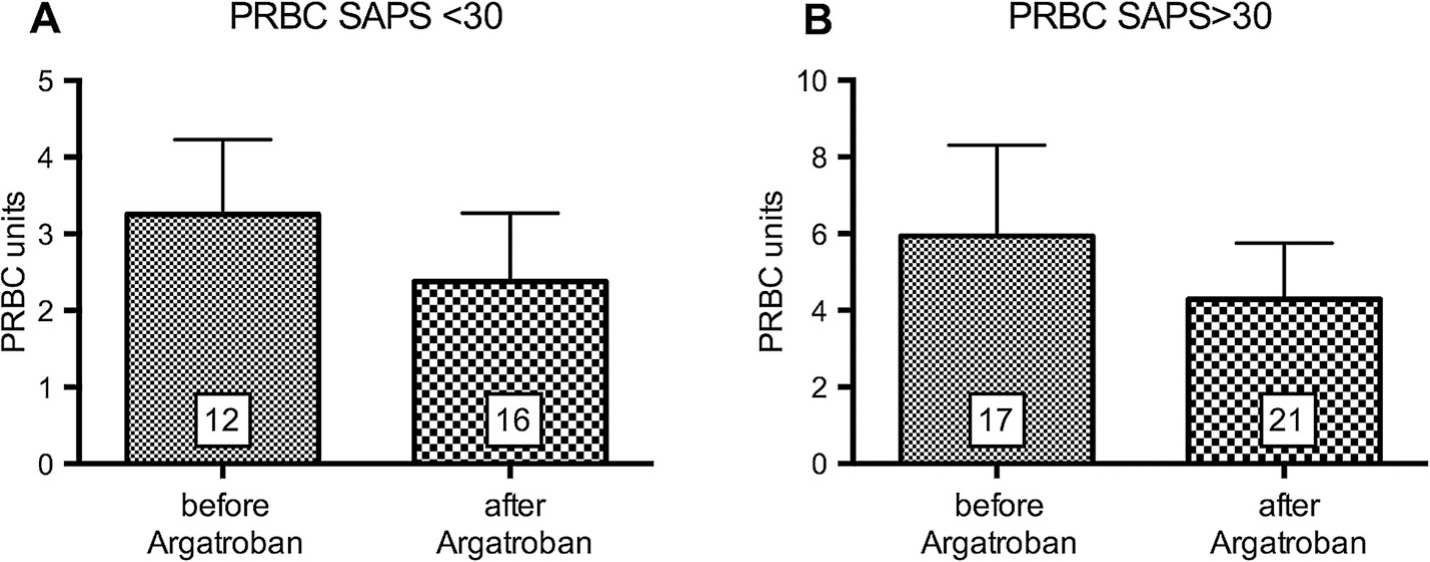
**Red blood cell transfusion under argatroban.** PRBC transfusion (mean ± SEM) in patients with **(A)** SAPS <30 and **(B)** SAPS >30 before and during argatroban therapy during ICU stay. *p* < 0.05.

The initial dosage of argatroban anticoagulation was 0.37 ± 0.069 mcg/kg/min in SAPS >30 and 0.35 ± 0.103 mcg/kg/min in SAPS <30 (Figure 1). Maintenance dosage was slightly reduced in SAPS >30 to 0.32 ± 0.067 mcg/kg/min to achieve aPTT-prolongation 1.5 to 3 times of the baseline. In SAPS <30, maintenance dosage was increased to 0.54 ± 0.248 mcg/kg/min to achieve effective anticoagulation (Figure 1, *p* > 0.05). The initial dosage in our study was below the manufacturer’s recommendations for critically ill patients with organ failure and patients with cardiac surgery (0.5 - 1.2 mcg/kg/min). There was no correlation between initial dosage (*r* = − 0.073) or maintenance dosage adjustment (*r* = − 0.326) and change in SAPS scores. Duration of argatroban therapy was 8.4 ± 2.11 days. Effective anticoagulation was monitored with aPTT which was prolonged 1.25-fold by 8.38 ± 2.0 s as compared with a mean aPTT prior to argatroban (45.12 ± 2.00 s, *n* = 41 vs. 36.02 ± 1.52 s, *n* = 32; *p* = <0.001). According to local hospital guidelines, anticoagulation for thromboembolic prophylaxis was efficient with an aPTT in the range of 35 to 40 s. In ten patients with a history of HIT, a baseline aPTT was not available before initiation of argatroban anticoagulation. None of the study patients developed a newly diagnosed thromboembolic complication under argatroban. Major bleeding was defined by a hemoglobin fall ≧ 2 g/dL and a transfusion of ≧ 2 PRBCs within 24 h. Argatroban infusion was discontinued temporarily in 16 patients for a total of 57 times. Reasons for discontinuation included diagnostic or surgical procedures (20/57; 35.1%), supratherapeutic aPTT (14/57; 24.6%) and bleeding (12/57; 21.1%). Hence, with this management and monitoring, red blood cell transfusion under argatroban therapy was not increased (Figure 2A and 2B, *p* > 0.05). Transfusions of fresh frozen plasma and platelets were not significantly affected by argatroban therapy.

### Elevated serum aminotransferases and argatroban

Moderate elevated serum aminotransferases (>3 times of the upper limit of normal) and increased total bilirubin were found in seven of the investigated patients. Initial dosage in these patients was slightly higher (0.40 ± 0.14 mcg/kg/min, *n* = 7) than in patients with normal hepatic parameters (0.36 ± 0.06 mcg/kg/min, *n* = 35; *p* = 0.7752). However, the initial dosage was slightly below the recommendation of 0.5 mcg/kg/min in patients with liver dysfunction (Child Pugh Class B). There was no difference in maintenance dosage during the rest of the ICU stay (0.42 ± 0.09 mcg/kg/min, *n* = 7 vs. 0.40 ± 0.12 mcg/kg/min, *n* = 35; *p* = 0.9490). In three patients with moderate elevated aminotransferases, argatroban therapy had to be discontinued due to hemoglobin fall or supratherapeutic aPTT. In these patients duration of discontinuation was disproportionately higher: 63.3% of the total interruption time was due to a fall in hemoglobin > 2 g/dL; 49.1% of total time of interruption in all patients due to supratherapeutic aPTT. Although, overall transfusion of blood components was unaffected by argatroban therapy in patients with moderate elevated aminotransferases, this data suggests a higher bleeding tendency in patients with moderate elevated aminotransferases.

### Impact of history of HIT

**Figure 3:**
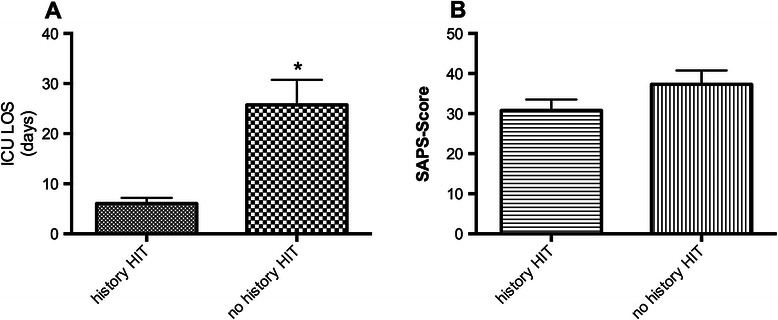
**Length of ICU stay and SAPS-score dependent of history of HIT. A)** ICU LOS and **B)** SAPS-score in patients with (*n* = 23) and without a history of HIT (*n* = 19). *p < 0.05.

**Figure 4:**
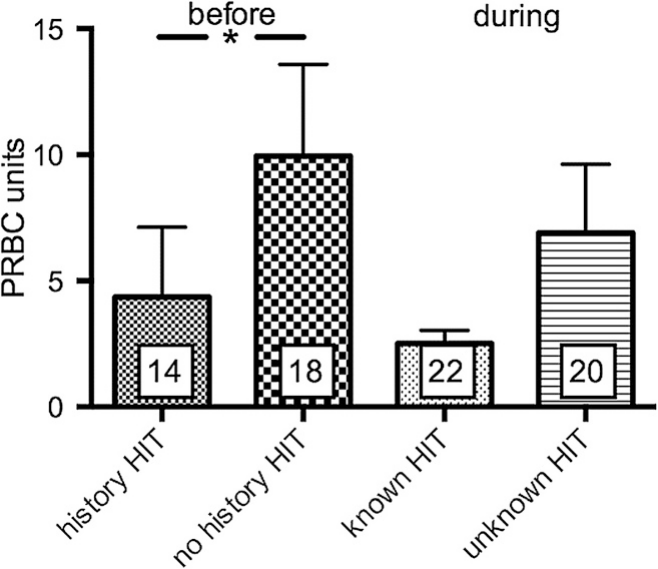
**PRBC before and during argatroban therapy in patients with and without a history of HIT.** * *p* < 0.05.

**Figure 5:**
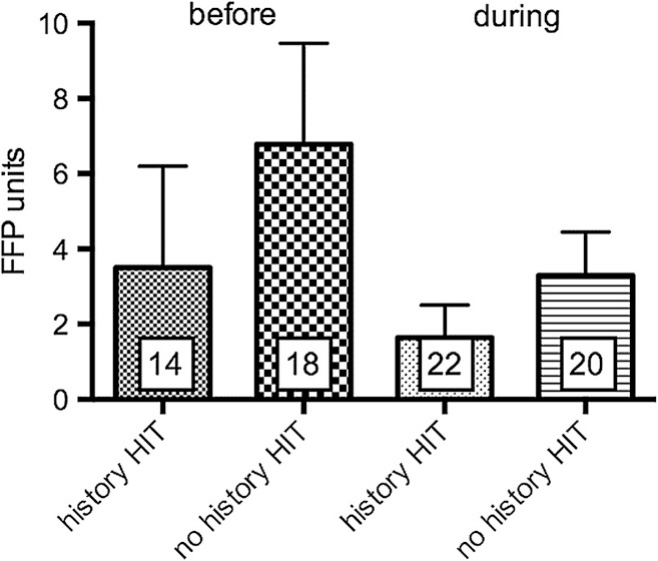
**Fresh frozen plasma (FFP) units consumption (mean ± SEM) before and during argatroban therapy in patients with known and unknown HIT.***p* < 0.05.

**Figure 6:**
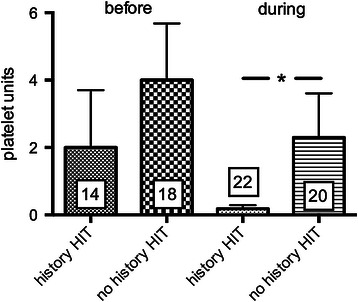
**Platelet units before and during argatroban therapy in patients with and without a history of HIT.** *p < 0.05.

There was a significant shorter length of stay in the ICU for patients with a history of HIT (6.0 ± 1.2 days, *n* = 23) compared to patients with no history of HIT (25.8 ± 5.0 days, *n* = 19; *p* < 0.001, Table 1, Figure 3A). Patients with no history of HIT had a slightly but not significantly higher SAPS-score (30.8 ± 2.7, *n* = 23 vs. 37.3 ± 3.5, *n* = 19; *p* = 0.1459, Figure 3B). ICU LOS was 7.5 ± 1.3 days before HIT diagnosis was established. Patients with a history of HIT received argatroban 13.22 ± 4.24 hours after admission to the ICU. Red blood cell transfusion was significantly less in patients with a history of HIT (4.4 ± 2.8 PRBC) before initiation of argatroban when compared with transfusion need of patients with no history of HIT (9.9 ± 3.6 PRBC) (Figure 4, *p* < 0.05). For FFP no statistical difference was found (Figure 5). Platelet transfusion was significantly lower during argatroban therapy in patients with a history of HIT (0.2 ± 0.1 platelet units) than in patients with no history of HIT (2.3 ± 1.3 platelet units) (Figure 6, *p* < 0.05).

### Cost calculations

Patients (*n* = 19) receiving heparin before HIT was suspected served as a comparison group for cost calculation of argatroban versus heparin administration. In this study population the mean dosage for heparin was 906.4 ± 186.5 IE/h or 21744 IE/d (*n* = 19), whereas the maintenance dosage of argatroban was 0.41 ± 0.10 mcg/kg/min (*n* = 42) or 49.6 mg/d (0.41 mcg × 84 kg × 1440 min). Considering these dosages and a wholesale price of 2.17 EUR for 25.000 IE heparin compared to 188.12 EUR for 250 mg (37.6 EUR per 50 mg) argatroban, the costs per day are about 17 times higher for argatroban administration than they are for heparin. In patients with no history of HIT, thus receiving heparin, transfusion requirements were higher than for patient with a history of HIT before initiation of argatroban therapy (4.4 ± 2.8 PRBC, *n* = 14 vs. 9.9 ± 3.6 PRBC, *n* = 18). Considering a PRBC unit price of 90 EUR, total costs for PRBC of 900 EUR for patients with no history of HIT under heparin versus 450 EUR for patients with a history of HIT have to be calculated. Patients had a mean ICU LOS of 7.5 ± 1.3 days before HIT suspicion was made. Assuming that these patients would have received argatroban in the first place instead of heparin, we estimate a total sum of 283.88 EUR for argatroban with a daily cost calculation of 37.60 EUR/d for argatroban multiplied with 7.5 days ICU LOS. This should be compared to 450 EUR additional costs for PRBC transfusions during heparin administration. Additional costs for laboratory testing (PF4 ELISA = 27.98 EUR, HIPAA = 52.46 EUR) would add up to a total of 364.32 EUR (Table 2).

**Table 2:** **Comparison of costs for heparin versus argatroban**

	Heparin	Argatroban
Mean dosage/ICU day	21744 IE	49.6 mg
Costs/ICU day	2.17 EUR	37.60 EUR
Total costs before HIT (mean ICU LOS before HIT suspicion = 7.5 days)	16.40 EUR	283.90 EUR
Costs for HIT diagnostics		27.98 EUR (PF4 ELISA)
		52.46 EUR (HIPAA)
PRBC transfusion before HIT suspicion	9.9 units	4.4 units
PRBC total costs before HIT (unit costs = 90 EUR)	900.00 EUR	450.00 EUR
Total costs for anticoagulation and transfusions	916.40 EUR	814.34 EUR
ICU LOS	26	6
Total costs of ICU stay (1050EUR/day)	27.000.00 EUR	6.300.00 EUR

An ICU LOS of 7.5 days and increased RBC transfusion with heparin anticoagulation were assumed. For further details see text.

Furthermore, patients with a history of HIT had a significant shorter ICU LOS. Assuming average costs of 1050.00 EUR per ICU day, total costs of ICU care in patients with a history of HIT would be 6 days × 1050 EUR = 6300 EUR versus 26 days × 1050 EUR = 27300 EUR in patients with no history of HIT, respectively resulting in a 77% cost reduction for patients with a history of HIT (Table 2).

## Discussion

The primary aims of this study were to investigate the safe dosage of argatroban in critically ill patients, thromboembolic complications and use of blood components. Furthermore, we analyzed the impact of a history of HIT on ICU LOS and the need for blood transfusions and the resulting economic consequences of argatroban versus heparin administration.

One striking finding of our study was that in our investigated population patients with a history of HIT had a much shorter ICU LOS when compared to patients with no history of HIT, regardless of SAPS score. The reason for a longer ICU LOS remains unclear. Patients with no history of HIT develop thrombocytopenia during their ICU stay because heparin is usually administered perioperatively. Thrombocytopenia itself is not a reason for an extended ICU stay. However, once suspicion of HIT arises, patients are closely monitored for thromboembolic events and argatroban therapy, which can be best established in the ICU. Red blood cell transfusion need was lower in patients with a history of HIT before anticoagulation with argatroban compared with patients with no history of HIT. There was also a difference for FFP and platelet transfusion, but the differences did not reach statistical significance. Platelet transfusions were less frequent in patient with a history of HIT during argatroban therapy. These findings imply that patients with no history of HIT are more prone to bleeding and have greater transfusion needs due to inadequate anticoagulation with heparin and thrombocytopenia. A recent study by Williamson et al. demonstrated that critically ill patients with thrombocytopenia have a higher risk of bleeding with subsequent transfusion need and increased ICU and hospital mortality [21]. The reported incidence of HIT is 0.3% in perioperative patients and associated with a 50% increased mortality [22]. Clinical development and suspicion of HIT, which is based mainly on significant thrombocytopenia, is usually established over the course of a couple of days. Once HIT is suspected, argatroban therapy is initiated. It has to be taken into consideration that platelet count may further decrease and thromboembolic complications may have already developed [5]. This may also affect transfusion needs during the remaining course of the ICU stay. In contrast, early screening of patients for HIT with a PF4 ELISA on admission to the ICU leads to overdiagnosis of HIT since it remains a clinical diagnosis [9]. Although a safety profile of argatroban in critically ill patients still needs to be established, bleeding complications are also common in patients on heparin [23]. Considering the fatality of thromboembolic complications due to HIT, general use of argatroban could be favorable. Furthermore, laboratory screening tests for HIT would be redundant if agratroban were be used in the first place.

Our retrospective analysis confirmed the current practice that the dosage should not be higher than 0.5 mcg/kg/min in critically ill patients, as recommended by the manufacturer. Moreover, a dosage below 0.4 mcg/kg/min can be considered safe in terms of thromboembolic events. Link et al. postulated a dosage calculation for argatroban in critically ill patients needing continuous renal replacement therapy using ICU scores such as APACHEII and SAPSII. A dose range of 0.5-1.2 mcg/kg/min would correspond to SAPS scores 30–52 according to a predicted dosage calculation [13]. In our patient population we did not find a correlation between argatroban dosage and SAPS scores. Maintenance dosage had to be slightly increased in SAPS <30 to achieve aPTT with 1.5 to 3-fold of baseline range. A SAPS score of 31 is consistent with severe illness and correlates with a predicted mortality of 11.7% [24,25]. In SAPS >30 critically ill patients in this study population, maintenance dosage was unchanged when compared to initial dosage. In the past, studies in critically ill patients have defined an argatroban dose range of lower than 0.5 mcg/kg/min [16,26,27]. These low dosages are not only safer for patients in terms of bleeding complications but are also more cost-effective for both blood component and argatroban consumption.

For economic considerations and cost calculations, patients with no history of HIT who received heparin before HIT suspicion was raised served as a comparison group. Although costs of argatroban administration were 17 times higher per day compared to heparin administration, these costs would be compensated by a reduced need for transfusions if argatroban was used as a first line anticoagulant in patients with no history of HIT. Interestingly, patients with no history of HIT had a significantly longer ICU LOS, which ultimately accounts for higher total costs for intensive care. Considering the devastating thromboembolic complications in 30% of cases associated with HIT, mortality rates up to 20% [28], an average cost-of-illness for confirmed HIT with thrombosis of 34155 Canadian Dollars (=22432 EUR/30847 USD in 2014) [29] and an incidence of up to 5% [30], alternative anticoagulants such as argatroban could have a better economic profile than heparin itself, despite argatroban’s higher market price.

As with all anticoagulants, major bleeding is one of the most common adverse events of argatroban therapy, but compared to a historical control bleeding complications are not increased [18,31]. Hepatic dysfunction and critical illness warrant dose adjustment as demonstrated by previous studies [16,27]. Doepker and coworkers identified argatroban-associated bleeding risk factors such as bilirubin >3 mg/dL, platelets <70 K/mcL and initial dosing body weight >90 kg [18]. Starting dose ranges vary between 0.2 mcg/kg/min in Beiderlinden’s study and 0.56 mcg/kg/min in Doepker’s study. Kiser et al. investigated a fixed dosage adjustment protocol to achieve a target aPTT. Although, their data suggest that 78% of their patient population was located in the ICU and had a dose regimen based on organ dysfunction, the mean initial dosage for argatroban was 1.5 mcg/kg/min and 1.3 mcg/kg/min for maintenance [32]. In the present retrospective study, we analyzed the safety of the dose ranges of argatroban administered to critically ill patients by assessing the need of transfusions before and during argatroban therapy. The overall need for blood component transfusions during argatroban therapy during ICU stay was not increased when compared with transfusion need before initiation of argatroban. However, argatroban therapy was temporarily discontinued when a major bleeding event occurred, during diagnostic and interventional procedures and with a supratherapeutic aPTT. While aPTT is the anticoagulation monitoring of choice for argatroban, incorporation in the daily ICU routine for HIT patients may be challenging due to multiple interruptions of therapy due to diagnostic or interventional procedures. Rotational thrombelastometry (ROTEM) evaluated in vitro shows a strong correlation between clotting time and aPTT. It could be a potential alternative for bedside monitoring of argatroban therapy [33]. However, major bleeding during argatroban therapy may still occur despite low dose regimen. Specifically, hepatic dysfunction (Child-Pugh class B) warrants dose reduction, since argatroban is metabolized in the liver by hydroxylation and aromatization of the 3-methyltetrahydroxyquinoline ring. In our study population, seven patients were identified with moderately elevated serum aminotransferases. Argatroban initial and maintenance dosage were below the manufacturer’s recommended dosage of 0.5 mcg/kg/min and did not differ when compared with all other study patients. Furthermore, the number of blood transfusions was not increased. However, argatroban therapy in patients with elevated aminotransferases had to be interrupted more often and for longer time intervals compared to all other patients. No thromboembolic complications were encountered in our patient population.

This study has some limitations. A heterogeneous surgical population was investigated in this retrospective analysis. Thus, bleeding risk factors are difficult to evaluate and argatroban therapy could have varying effects on overall bleeding. APTT and interruption of argatroban may not reflect a direct anticoagulatory effect of argatroban. Minimum aPTT range to prevent thrombembolic complications under argatroban was not evaluated. Furthermore, this study lacks a historical control group due to heterogeneity of the investigated patients. However, it was not the aim of this study to directly compare argatroban with heparin in terms of bleeding risk. We compared parameters before and after argatroban in patients who had no history of HIT, thus receiving heparin before HIT was suspected. The transfusion requirement data was not corrected for the time before initiation of argatroban. It has to be considered that this comparison group had a higher risk of bleeding with respect to HIT while heparin was still administered requiring more PRBC transfusions.

## Conclusion

Low dosage of argatroban for critically ill patients is safe with respect to thromboembolic complications. Clinical monitoring of bleeding in the surgical population remains the most important parameter for adequate argatroban dosing while close aPTT monitoring remains a challenge for the daily ICU routine. Our data suggests that patients with new onset of HIT have an overall longer ICU stay and higher risk of bleeding. Cost calculations favor the use of argatroban in patients with no history of HIT necessitating laboratory screening. This should be investigated further.
